# SEC-based isolation of phosphatidylserine-liposomes from biological samples for preclinical studies

**DOI:** 10.1007/s00216-026-06436-y

**Published:** 2026-03-16

**Authors:** Oumaima El Ouahabi, Montserrat Mancera-Arteu, Ángeles López-Godoy, Irene Latorre, Míriam Salvadó, Bruna Barneda-Zahonero, Sílvia Rodríguez-Vidal, Victoria Sanz-Nebot

**Affiliations:** 1 Department of Chemical Engineering and Analytical Chemistry, Institute for Research on Nutrition and Food Safety (INSA·UB), University of Barcelona, Martí i Franquès 1-11, 08028 Barcelona, Spain; 2 Ahead Therapeutics SL, Barcelona, Spain

**Keywords:** Liposomes, Phosphatidylserine, Liquid chromatography, Size exclusion chromatography, Mass spectrometry, Serum

## Abstract

Autoimmune diseases represent a significant clinical challenge due to their high prevalence, chronic progression, and profound impact on patients’ quality of life. Despite advances in understanding their pathophysiological mechanisms, in most cases, only palliative treatments are available. In this regard, a novel therapeutic approach is under development, using phosphatidylserine-liposomes (PS-Liposomes) encapsulating specific antigenic peptides that trigger the autoimmune attack. By varying the encapsulated antigen, this method offers the potential to treat different autoimmune diseases. The development of robust bioanalytical methods to characterize PS-Liposomes in biological fluids is of great importance to evaluate their efficacy and safety in preclinical trials. This presents a significant challenge due to the presence of extracellular vesicles and proteins in blood with similar size and composition to PS-Liposomes. In this study, size exclusion chromatography (SEC) was assessed for the isolation of PS-Liposomes encapsulating acetylcholine receptor peptide (AChR), intended for myasthenia gravis treatment, from minipig serum samples. A home-packed column filled with Sepharose 2B-CL resin and two commercial columns with agarose resin (qEV 35 nm and qEV 70 nm) were tested by using fluorescently labelled PS-Liposomes. Fractions containing PS-Liposomes were monitored by flow cytometry and qEV columns were selected for further experiments. For PS-liposome preconcentration of the obtained fractions, two different methodologies were also evaluated (Centrisart filters and qEV kit). Lipid and antigenic peptide recoveries obtained for each method were determined by HPLC-ELSD and HPLC-MS, and the optimum isolation and preconcentration approach was selected. Finally, the established methodology was successfully applied to spiked minipig serum with unlabelled PS-Liposomes, and the preservation of structural integrity after isolation was demonstrated by nanoparticle tracking analysis (NTA) and scanning electron microscopy (SEM).

**Graphical abstract:**

**Figure:**
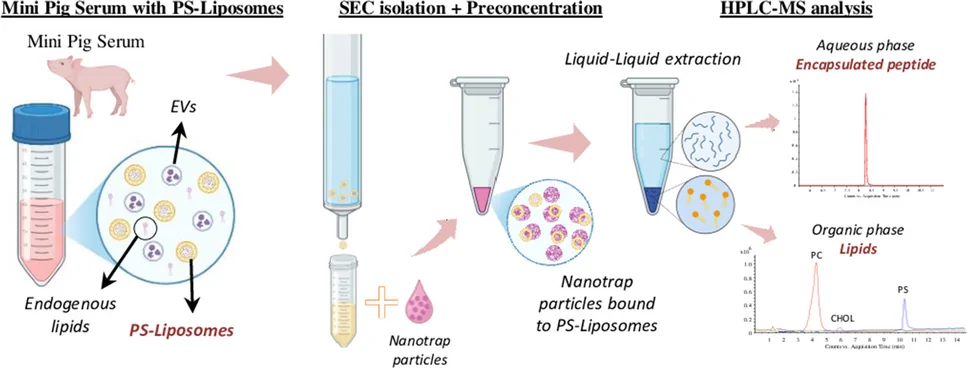

**Supplementary Information:**

The online version contains supplementary material available at https://doi.org/10.1007/s00216-026-06436-y.

## Introduction

Liposomes are self-assembled vesicular structures composed of phospholipids organized into a bilayer or concentric multilamellar shells surrounding an aqueous core [1, 2]. Owing to their ability to encapsulate both hydrophilic and hydrophobic molecules and its capacity to transport bioactive compounds, liposomes have become an important drug delivery system [3, 4]. A novel therapeutic strategy based on phosphatidylserine-rich liposomes (PS-Liposomes) has emerged for modulating autoimmune responses. Autoimmune diseases (AIDs) are chronic, multifactorial disorders of unclear etiology, for which available treatments remain predominantly palliative [5,. Unlike conventional therapies, PS-Liposomes encapsulating the antigen responsible for triggering the autoimmune response aim to induce antigen-specific immune tolerance by mimicking apoptotic cells. In this way, the immune system is reprogrammed, and the immune attack is stopped [7–10]. Additionally, by simply modifying the encapsulated antigenic peptide or protein, this strategy can be used for the treatment of different autoimmune diseases. PS-liposome-based therapy has already shown promising results in preclinical studies of different pathologies including type I diabetes, multiple sclerosis, rheumatoid arthritis, and myasthenia gravis [7, 11].

The development of robust bioanalytical methods is essential to isolate and characterize PS-Liposomes from biological fluids, thereby supporting preclinical pharmacokinetic and toxicology studies. However, isolating PS-Liposomes from biological matrices remains challenging due to the presence of extracellular vesicles (EVs) and other lipid aggregates with similar sizes and compositions to PS-Liposomes [11]. The isolation of EVs from biological fluids has been widely studied using different methodologies. Centrifugation, ultrafiltration, precipitation with several agents, size exclusion chromatography (SEC), and immunoaffinity capture-based techniques were described for this purpose [12–16]. Moreover, different commercial columns and kits for EVs isolation are currently available [17]. In contrast, protocols for liposome isolation from biological fluids are scarce. Only SEC, ultrafiltration, and centrifugation have been previously described for liposome isolation from plasma or serum samples [18–21]. However, in centrifugation and ultrafiltration approaches, liposomes are subjected to high centrifugal forces, which can induce their structural deformation and formation of aggregates. Otherwise, SEC-based isolation of liposomes can be considered a promising approach as it enables particle separation across a relevant size range while preserving the particle’s structure, integrity, and biological function. To the best of our knowledge, no previous studies reported a quantitative workflow specifically designed for the isolation and comprehensive characterization of polydisperse liposome formulations from serum samples.

In this work, SEC was studied for PS-liposome isolation from minipig serum samples, given the method’s capacity to maintain particle’s integrity and inherently heterogenous size distribution of PS-Liposomes. An in-house packed SEC column with Sepharose CL-2B (100 nm pore size) was compared with two commercial agarose-based SEC columns (qEV 35 and qEV 70, with pore sizes of 35 nm and 70 nm, respectively) by using fluorescently labelled PS-Liposomes, encapsulating acetylcholine receptor peptide (AChR) for myasthenia gravis (MG) treatment. Collected fractions were monitored by flow cytometry allowing to select qEV columns for further experiments. For PS-liposome preconcentration of the obtained SEC fractions, two different preconcentration strategies were tested: Centrisart filters and a commercial qEV concentration kit (qEV kit). This kit was intended for EV isolation from blood samples. Recoveries for lipids were firstly determined by high-performance liquid chromatography with evaporative light scattering detector (HPLC-ELSD), after liposome lysis and components separation by applying a liquid-liquid extraction protocol [23]. However, interferences from blood lipids in the analysis prompted the development of a new method by high-performance liquid chromatography coupled to mass spectrometry (HPLC-MS) for accurate identification of PS-liposome lipids. Recoveries for encapsulated antigenic peptide were also evaluated by HPLC-MS for each applied method. The results obtained for both lipids and antigen demonstrated that the qEV 35 nm column, combined with preconcentration using the qEV kit, was the optimal condition for isolating PS-Liposomes from minipig serum samples. Finally, the methodology was successfully applied to non-labelled PS-Liposomes, yielding consistent recoveries for both lipids and antigenic peptide, which demonstrates its applicability for future pharmacokinetic and toxicology studies. The preservation of structural integrity and size distribution of the isolated PS-Liposomes was also studied by nanoparticle tracking analysis (NTA) and scanning electron microscopy (SEM).

## Experimental

### Materials and solutions

Chloroform (CHCl_3_), trifluoracetic acid (TFA), acetonitrile (ACN), hydrochloric acid (HCl), sodium hydroxide (NaOH), and sodium azide were purchased from Sigma-Aldrich (Darmstadt, Germany). Methanol (MeOH) was obtained from Honeywell (Seelze, Germany). Ethanol was provided by Panreac AppliChem (Barcelona, Spain). Dulbecco’s phosphate buffer saline (DPBS 1×) was obtained from Cytiva (Logan, UT). Phosphatidylcholine (PC; 1,2-dimyristoyl-sn-glycero-3-phosphocholine 14:0, MW 677.94 g/mol) and phosphatidylserine (PS; 1,2-dioleoyl-sn-glycero-3-phospho-l-serine 18:1, MW 810.04 g/mol) (analytical standards, ≥ 99.0%) were purchased from Avanti Polar Lipids (Alabaster, AL, USA). Cholesterol (United States Pharmacopeia (USP) reference standard quality, ≥ 99.0%) and 3-hexanoyl-NBD cholesterol (≥ 98.0%) were obtained from Merck (Darmstadt, Germany) and Cayman Chemical (MI, USA), respectively. The chemical structures of the PC, PS, and cholesterol species used in this study are provided in the Supplementary Material (Figure S-1). Acetylcholine receptor (AChR) antigenic peptide (MKLGTWTYDGSVVAINPESD-amide, 20 amino acids) was provided by the Peptide Synthesis Facility corresponding to the Department of Medicine and Life Sciences of the Pompeu Fabra University (Barcelona, Spain), with a purity exceeding the 90%.

Individual standard stock solutions of PC, PS, and cholesterol were prepared in CHCl₃ at a concentration of 10 mg/mL and stored at −80 °C until use. Calibration standards were prepared by diluting these standard stock solutions with chloroform to the calibration range of 50–500 mg/L. The standard stock solution of AChR peptide was prepared in water at a concentration of 1000 mg/L and stored at −20 °C until its use. Calibration standards for the peptide were then prepared from the standard stock solution by further diluting with water to the concentration range of 2.5–100 mg/L.

Minipig blood samples were provided by SpeciPig SL (Barcelona, Spain). The blood samples were allowed to stand at room temperature for 2 h to complete coagulation and then centrifuged at 1500 × g for 10 min to separate the clot. The supernatant was carefully recovered by pipetting, and the serum was aliquoted into 3-mL vials. The aliquots were stored in the freezer at −20 °C until use.

### Liposome formulation manufacturing

PS-liposome formulations were manufactured by a solvent-injection method in a cleanroom facility at Ahead Therapeutics (Arboç, Catalonia, Spain). Liposomes were composed of phosphatidylcholine (PC, Lipoid), phosphatidylserine (PS, Lipoid), and cholesterol (CHOL, Merck). Lyophilized lipids were dissolved in ether at 55 °C, and the lipid solution was rapidly injected into the antigenic peptide solution (dissolved in DPBS). Finally, PS-Liposomes were extruded using a Lipex® Extruder (Evonik) and dialyzed for 24 h, resulting in a milky homogeneous suspension. For the manufacture of fluorescent PS-Liposomes, 0.15% molar of 3-hexanoyl-nitrobenzoxadiazole (NBD)-tagged cholesterol in ether were added to PC, PS, and CHOL solution in ether, which was rapidly injected into the DPBS solution containing the antigenic peptide.

### PS-liposome isolation and preconcentration

Size exclusion chromatography experiments were performed at room’s temperature using a home-packed column filled with Sepharose CL-2B resin (Sigma-Aldrich) and two commercial columns with enhanced agarose resin of different pore sizes (qEVoriginal Column 35 and 70 nm, Izon Science Limited).

For home-packed Sepharose column, the Sepharose CL-2B resin was firstly washed with DPBS, using a 1:1 volume ratio of resin to buffer, to remove storage buffer (20% v/v ethanol) prior to packing. The washed slurry was transferred to Econo-Column Chromatography Columns, 1.5 × 15 cm (BioRad) until the desired bed volume was reached (30 mL). The resin was let to settle overnight and the top frit was then placed gently on the resin without compressing. For sample purification, the column is firstly equilibrated at room temperature and conditioned with 30 mL of DPBS. Then, five hundred microliters of sample (i.e., PS-liposome solution or spiked minipig serum at a TLC of 1 mM) was loaded onto the column. The sample was allowed to enter the column completely, and the column was topped up with DPBS. Immediately, 24 sequential fractions of 0.5 mL were collected in clean tubes. Before loading the next sample, the column was washed with 30 mL of DPBS. The column was operated at a flow rate of 0.5 mL/min by using a low-pressure flow adaptor. After use, the column was stored at 4 °C in 20% (v/v) ethanol solution.

For qEVoriginal columns, manufacturer instructions were followed. Briefly, the column is firstly equilibrated at room temperature and conditioned with 17 mL of DPBS to eliminate storage buffer (0.05% w/v sodium azide). Then, five hundred microliters of sample (i.e., PS-liposome solution or spiked minipig serum at a TLC of 1 mM) was loaded onto the column. The sample was allowed to enter the column completely, and the column was topped up with DPBS. Twenty-four sequential fractions of 0.5 mL were then collected in clean tubes. Under the optimized conditions, the first 1.5 mL of eluted buffer was discarded, and the subsequent 6.5 mL of eluate, containing mainly the PS-Liposomes, was selected for further analysis. Before loading the next sample, the column was rinsed with 8.5 mL of 0.5 M NaOH to remove residual proteins and flushed with 17 mL of DPBS. For column storage, 17 mL of DPBS containing 0.05% w/v sodium azide was flushed, and the column was stored at 4 °C until its next use.

After collecting the fractions, a preconcentration step was necessary to reduce the eluted volume and thereby increase PS-liposome concentration. For this purpose, two different methodologies were evaluated (qEV kit and Centrisart filters). For qEV concentration kit (Izon Science Limited), one hundred microliters of particles solution was added to 1900 µL of the collected fractions. The mixture was incubated with gentle rotation for 1 h to ensure an efficient binding of PS-Liposomes to the qEV kit particles. Afterwards, the incubated sample was submitted to centrifugation at 16,800 × g for 10 min at room temperature, and the supernatant was carefully removed. The pellet, containing PS-Liposomes, was finally resuspended in 190 µL of DPBS for subsequent analysis. In the case of Centrisart 300 kDa filters (Sartorius AG), 1900 µL of the collected fractions was loaded to the centrifuge tube (outer tube), after removing the floater (inner tube). Then, the floater was reinserted, and the device was allowed to stand for 5 min to ensure complete membrane wetting. After centrifugation at 2500 × g for 30 min at room temperature, the floater was rapidly removed, and the concentrated PS-Liposomes were obtained in the centrifuge tube. The final volume was adjusted to 190 µL by adding DPBS if necessary.

Finally, liquid-liquid extraction protocol previously established [23] was applied to achieve liposome lysis and extract/separate PS-liposome components (i.e., encapsulated peptide and lipids). Briefly, 62.5 µL of chloroform and 125 µL of methanol were added to 50 µL of liposomal formulation, allowing liposome lysis. The mixture was vortexed, and 125 µL of liposome sample and 62.5 µL of chloroform were added. The mixture was then centrifuged at 12,000 rpm for 10 min at room temperature to ensure separation of the two phases. For HPLC-ELSD analysis, 100 µL of the lower organic phase was transferred to a clean HPLC vial. Linearity range was established between 20 and 200 mg/L for all lipids. In the case of HPLC-MS analysis, [23] 100 µL of the lower organic phase was diluted with chloroform to fall within the calibration range (5–25 mg/L) and transferred to a clean HPLC vial for lipid quantification. Likewise, 100 µL of the upper aqueous phase, which contains the extracted antigenic peptide, was transferred to a clean HPLC vial for peptide quantification.

### Lipid quantification by HPLC-ELSD and HPLC-MS

Lipid separation and quantification experiments were conducted by both HPLC-ELSD and HPLC-MS using a Luna C_18_(2) column (5 µm particle diameter, 100 Å pore diameter, 150 × 2 mm total length (L_T_) × internal diameter (ID), Phenomenex).

For HPLC-ELSD, a 2790/2975 liquid chromatograph (Waters Corporation, Milford, MA, USA) coupled to a SEDEX 45 ELSD detector (Sedex Sedere, Alfortville, France) was used following the conditions previously described [23]. Briefly, experiments were conducted at 35 °C with gradient elution at a flow rate of 0.5 mL/min and a sample injection volume of 10 µL. The eluting solvents were water (A) and MeOH with 0.1% (v/v) TFA (B). The optimized gradient for solvent B was as follows: from 85 to 100% (v/v) (0–10 min), 100% (v/v) (10–15 min), from 100 to 85% (v/v) (15–18 min), and 85% (v/v) (18–25 min). The ELSD parameters were set to 40 °C for nebulizer temperature, 2 bars for inert gas flow, and 9 for PMT gain. For HPLC-MS analysis, a 1260 Infinity liquid chromatograph (Agilent Technologies, Waldbronn, Germany) coupled to a 6220 orthogonal acceleration (oa)-TOF LC/MS mass spectrometer with an orthogonal G1385−44300 interface (Agilent Technologies) was used. Experiments were conducted at 35 °C with gradient elution at a flow rate of 0.35 mL/min and injecting 10 μL of each sample. The mobile phase used was composed of A: MeOH:H₂O (95:5) and B: isopropanol (IPA) with 0.5% (v/v) formic acid (FA). The optimized gradient for solvent B was as follows: from 15 to 20% (v/v) (0–5 min), from 20 to 50% (v/v) (5–6 min), 50% (v/v) (6–12 min), from 50 to 15% (v/v) (12–13 min) and 15% (v/v) (13–20 min). The mass spectrometer was equipped with a dual-nebulizer ESI source, and the orthogonal nebulizer was used for the LC-TOF-MS experiments; the second nebulizer, which is generally used to introduce the internal reference mass standard solution, was disabled to avoid interferences. Full-scan acquisition mode was applied, and positive electrospray ionization (ESI+) was used for all analytes. Tuning and calibration of the mass spectrometer were carried out in accordance with the manufacturer’s instructions. The measurement parameters were subsequently fine-tuned by direct infusion of the lipid standards. Optimal measurement parameters in positive ion mode are the following: capillary voltage 3500 V, drying gas (N₂) flow rate 8 L/min, drying gas (N₂) temperature 350 °C, nebulizer gas (N₂) 30 psig, fragmentor voltage 150 V, skimmer voltage 60 V, and OCT 1 RF Vpp voltage 250 V. Data were acquired in profile, recording 1 spectrum per second between 100 and 3200 m/z, at the highest resolution mode (4 GHz). Chemstation software (Agilent Technologies) and MassHunter workstation software (Agilent Technologies) were used for control, data acquisition, and processing of the HPLC and MS instruments, respectively. Quantification of PS, PC, cholesterol, and the AChR peptide was performed using extracted ion chromatograms (EICs) of their exact masses in high-resolution MS mode. The specific ions and exact m/z values used are listed in Supplementary Table S-1.

### Peptide quantification by HPLC-MS

Separation and quantification of antigenic peptide was performed using a 1260 Infinity liquid chromatograph (Agilent Technologies, Waldbronn, Germany) coupled to a 6220 oa-TOF LC/MS mass spectrometer with an orthogonal G1385−44300 interface (Agilent Technologies). Chromatographic separation was performed using a Zorbax 300SB-C_3_ column (3.5 µm particle diameter, 300 Å pore diameter, 150 × 4.6 mm total length (LT) × internal diameter (ID), Agilent Technologies), with gradient elution at a flow rate of 0.5 mL/min. The solvents used were water (A) and acetonitrile (B), both containing 0.1% (v/v) FA. The optimized gradient for solvent B was as follows: from 5 to 100% (v/v) (0–10 min), 100% (v/v) (10–15 min), from 100 to 5% (v/v) (15–18 min) and 5% (v/v) (18–25 min). Injection volume was set to 20 µL. Measurement parameters for the MS are the following: capillary voltage 4000 V, drying gas (N₂) flow rate 8 L/min, drying gas (N₂) temperature 350 °C, nebulizer gas (N₂) 30 psig, fragmentor voltage 325 V, skimmer voltage 80 V, and OCT 1 RF Vpp voltage 250 V. Data were acquired in profile, recording 1 spectrum per second between 100 and 3200 m/z, at the highest resolution mode (4 GHz). Chemstation software (Agilent Technologies) and MassHunter workstation software (Agilent Technologies) were used for control, data acquisition, and processing of the HPLC and MS instruments, respectively.

The encapsulated AChR peptide content was determined by quantifying free peptide, after ultracentrifugation at 12,000 rpm for 40 min, and total peptide, after liposome lysis using the liquid-liquid extraction procedure described above [23]. The difference between total and free peptide concentrations was used to calculate the amount of encapsulated peptide.

### Flow cytometry, NTA, and SEM

The protocols for flow cytometry, nanoparticle tracking analysis (NTA), and scanning electron microscopy (SEM) are provided in the Supplementary Material.

## Results and discussion

### Optimization of PS-liposome isolation and preconcentration. Analysis by HPLC-ELSD

**Fig. 1:**
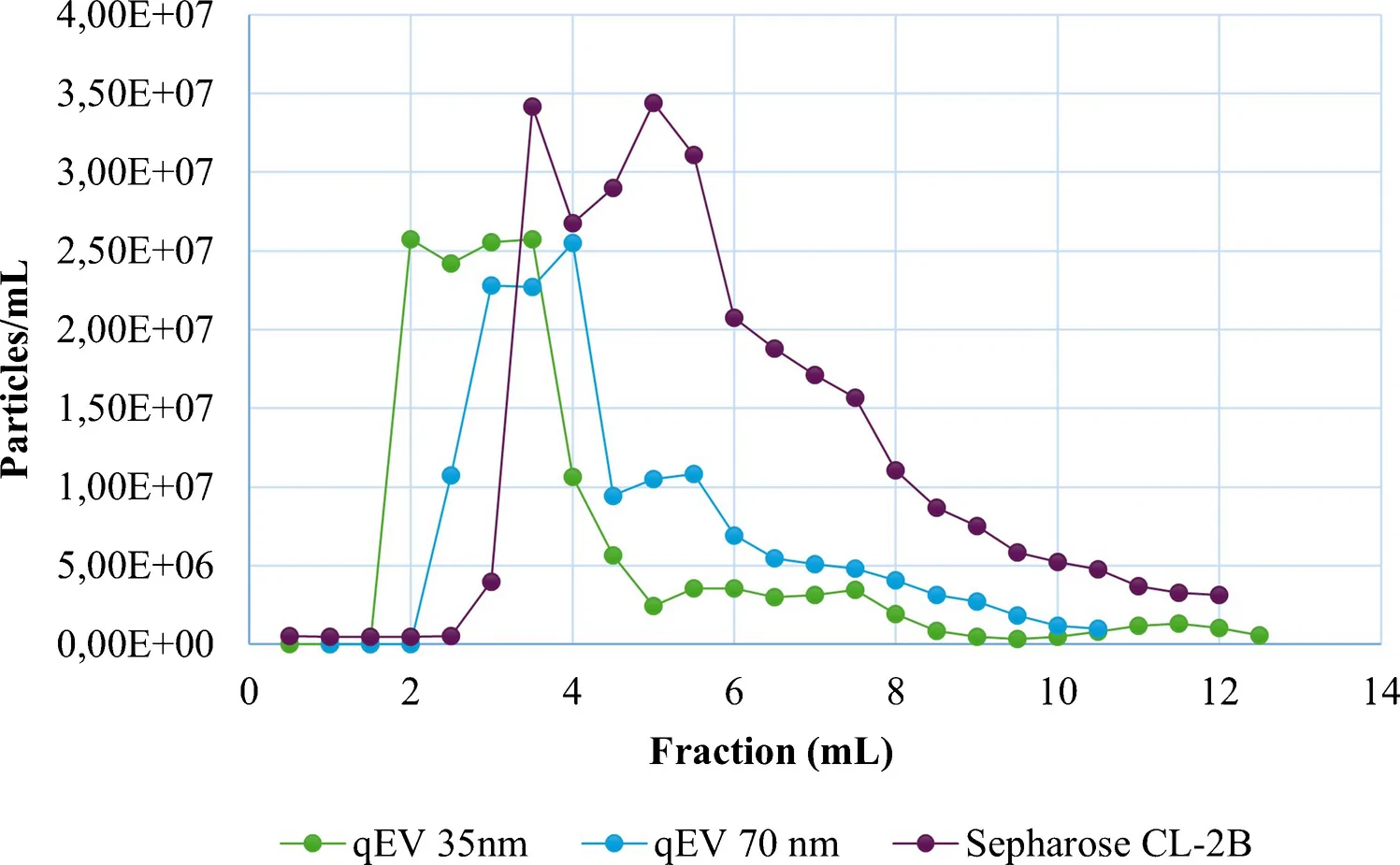
Graphic representation of the particle’s concentration obtained in each 0.5 mL collected fraction after SEC isolation of labelled PS-liposome formulation encapsulating AChR peptide for all columns tested. Each data point corresponds to an individual collection fraction. Fluorescent particles of known concentration were used as a standard to estimate liposome concentration

The similarity in composition and size between PS-Liposomes and other extracellular vesicles poses a challenge for their isolation from serum samples. To address this, size exclusion chromatography (SEC) was selected for the purification of PS-Liposomes from serum, as it enables effective size-based separation across distinct fractions. Firstly, different SEC columns were evaluated using PS-Liposomes fluorescently labelled with NBD-cholesterol. A home-packed column filled with Sepharose 2B-CL resin and two commercial columns with enhanced agarose resin of different pore sizes (qEVoriginal Column 70 and 35 nm) were compared. Sepharose 2B-CL was assessed as it was previously used for liposome isolation [22]. Otherwise, qEV columns, designed for EVs isolation, were also evaluated due to the membrane charge and size similarities between exosomes and PS-Liposomes, as well as their reported ability to effectively eliminate HDL lipoproteins and free proteins. A PS-liposome formulation encapsulating AChR peptide (Batch 1) at a total lipid concentration (TLC) of 1 mmol/L (600 mg/L) was purified by using each SEC column. This PS-liposome formulation is intended for the treatment of myasthenia gravis (MG) and was used as a model for SEC evaluation and optimization. After each SEC purification, 24 fractions of 500 µL each were collected and analyzed by flow cytometry using fluorescence to determine the fractions in which the liposomes eluted. Figure 1 shows the graphic representation of the particle’s concentration obtained in each fraction for all SEC columns tested. Fluorescent particles of known concentration were used as a standard to estimate liposome concentration. The results showed that with the Sepharose column, many more fractions had to be collected to recover main eluted liposomes, yielding a total volume of 12 mL compared to the around 6 mL needed with the qEV columns (Fig. 1). Therefore, agarose qEV columns were selected for further experiments.

In order to determine the most suitable qEV column for PS-liposome isolation, the lipids recovery of both columns was evaluated. For this purpose, fractions 4 to 16, which correspond to 2–8 mL, were selected as they allowed the collection of the highest number of liposomes in the smallest volume (Fig. 1). The selected fractions were mixed, and the resulting solution was preconcentrated tenfold using nanotrap particles commercial kit (qEV kit), described for extracellular vesicles enrichment. Subsequently, liposome disruption was achieved using a liquid-liquid extraction protocol, and the organic phase containing the lipids was analyzed by liquid chromatography with an evaporative light scattering detector (HPLC-ELSD), as previously described. A calibration curve ranging from 20 to 200 mg/L was established for each lipid. By way of example, Figure S-2 shows the chromatogram obtained by HPLC-ELSD for the analysis of the three lipids that conform the PS-liposome membrane (PC, CHOL, and PS) at a concentration of 200 mg/L each. The recoveries obtained for each lipid using the different agarose qEV columns are presented in Table 1. The recoveries were calculated relative to the total lipid concentration (TLC) determined in the PS-liposome formulation before SEC purification. Comparing the recovery values obtained using both columns, it was observed that the qEV 35 nm column yielded slightly higher recovery rates and improved reproducibility compared to the qEV 70 nm column. Based on these findings, the qEV 35 nm column was selected for the following experiments. In addition, it was evaluated whether liposomes eluting in the later fractions contributed significantly to overall lipid recovery. For this purpose, two aliquots of the same PS-liposome formulation (Batch 1) at a TLC of 1 mmol/L were purified by qEV 35 nm column. In the first replicate, only the previously selected fractions (4–16) were collected, while in the second one, later fractions were also included (4–24). Subsequently, the selected fractions were mixed and preconcentrated by qEV kit. Liposome disruption was achieved with the liquid-liquid extraction method and the organic phase containing the lipids was analyzed by HPLC-ELSD. The analysis showed very similar recoveries in both cases (Table S-2), calculated relative to the determined TLC before SEC purification. Thus, liposomes in fractions 17–24 were discarded, and fractions 4–16 were considered representative and collected for subsequent analyses.

**Table 1:** Lipid recovery results obtained by HPLC-ELSD for labelled PS-liposome formulation encapsulating AChR peptide after isolation with both agarose qEV columns and preconcentration using different methods (qEV kit or Centrisart filters)

Recovery (%)	qEV 35 nm column + qEV kit preconc	qEV 70 nm column + qEV kit preconc	qEV 35 nm column + Centrisart filters preconc
PC	36 ± 2	29 ± 4	52 ± 3
PS	29 ± 2	19 ± 3	52 ± 2
CHOL	36 ± 3	28 ± 3	53 ± 3
TLC	34 ± 2	25 ± 3	55 ± 3

With the aim of improving the recoveries for PS-liposome isolation, an alternative preconcentration method was evaluated by using Centrisart ultrafiltration filters. Thus, using the qEV 35 nm column to purify the PS-liposome formulation, preconcentration methods using either the qEV kit or Centrisart filter were compared. After applying the liquid-liquid extraction protocol to each sample, the obtained organic phases were analyzed by HPLC-ELSD. As can be observed in Table 1, Centrisart filters allowed for better lipid recoveries, with similar reproducibility in both cases. Since the goal of this work is to apply the methodology to blood serum samples, the effectiveness of both methods was also evaluated using firstly a minipig serum blank, which was purified by the qEV 35 nm column and preconcentrated separately by using each methodology (Centrisart or qEV kit). In this way, the possible interference of lipids from extracellular vesicles or other serum components in the subsequent HPLC-ELSD analysis was evaluated. Figure S-3 shows the chromatograms obtained by HPLC-ELSD for the analysis of each organic phase, after applying the liquid-liquid extraction method. As can be observed, after preconcentration with the qEV kit, only a very low-abundance peak is observed in the region where the lipids from PS-Liposomes elute. In contrast, a contribution of several abundant peaks in the same region was obtained after Centrisart preconcentration, which would interfere in the quantification of the target lipids by HPLC-ELSD. Therefore, although Centrisart filters provided better recoveries, they were discarded for serum analysis by HPLC-ELSD.

Furthermore, in order to evaluate possible concentration-dependent recoveries, different liposome concentrations were tested. It is important to note that very low PS-liposome concentrations are expected in pharmacokinetic and toxicology studies, owing to their specific mechanism of action that leads to rapid phagocytosis of PS-Liposomes within minutes. Thus, the isolation and preconcentration methodology using the qEV 35 nm column and the qEV kit, respectively, was applied to Batch 1 at different TLC concentrations (0.2, 0.5, 1, and 2 mmol/L), prepared by a serial dilution of the initial formulation. These concentrations were calculated as the sum of the molar concentration obtained for each individual lipid component. Liquid-liquid extraction method was then used for liposome lysis in each eluate, and the obtained organic phase was analyzed by HPLC-ELSD. As a result, lipids were successfully detected and quantified at all concentrations, demonstrating good method performance also at very low liposome concentrations. However, as shown in Table S-3, certain concentration-dependent recoveries were obtained. Samples with TLC concentrations below 1 mmol/L showed lower recoveries compared to the 1 and 2 mmol/L samples, which exhibited similar recoveries.

In order to assess the isolation performance of the method, spiked minipig serum samples before and after isolation by qEV 35 nm only, qEV kit only, and qEV 35 nm + qEV kit method were analyzed and compared. After liquid-liquid extraction, the aqueous phases, containing the peptides, and the organic phases, containing the lipids, obtained in each case were analyzed by HPLC-UV and HPLC-ELSD, respectively. As shown in Supplementary Figures S-4 and S-5, a very high contribution of serum-derived components was obtained when no isolation is applied. Otherwise, although the use of only isolation by qEV 35 nm column or qEV kit significantly reduces contribution of these compounds, the combination of qEV 35 nm column + qEV kit provided the best results considering both selectivity and sensitivity,

### Optimization of PS-liposome isolation and preconcentration. Analysis by HPLC-MS

In order to reduce matrix-related interferences in the analysis, it was decided to transfer the HPLC-ELSD method to liquid chromatography coupled to mass spectrometry (HPLC-MS). This transition was also crucial to achieve lower limits of detection needed for future pharmacokinetic studies. Thus, a new HPLC-MS method was developed for the analysis of PC, PS, and cholesterol from PS-Liposomes using the same Luna C18 column. For this purpose, different mobile phases and gradients were evaluated. TFA was replaced in all cases by FA due to its incompatibility with mass spectrometry (MS). Using the same mobile phase as in HPLC-ELSD but replacing TFA with FA did not result in proper PS elution, although different gradients were tested (data not shown). Similarly, increasing the FA content also failed to improve PS elution, leading to the evaluation of a different mobile phase previously described for lipid analysis by MS [24]. As a result, a mobile phase composed of A: MeOH:H_2_O (95:5) and B: 0.5% FA in IPA was established for the proper elution and separation of PC, PS, and cholesterol from PS-Liposomes by HPLC-MS. By way of example, the extracted ion chromatograms (EICs) obtained by HPLC-MS for the analysis of a mixture of the three lipids at a concentration of 25 mg/L each under the optimized conditions are shown in Figure S-6.

**Fig. 2:**
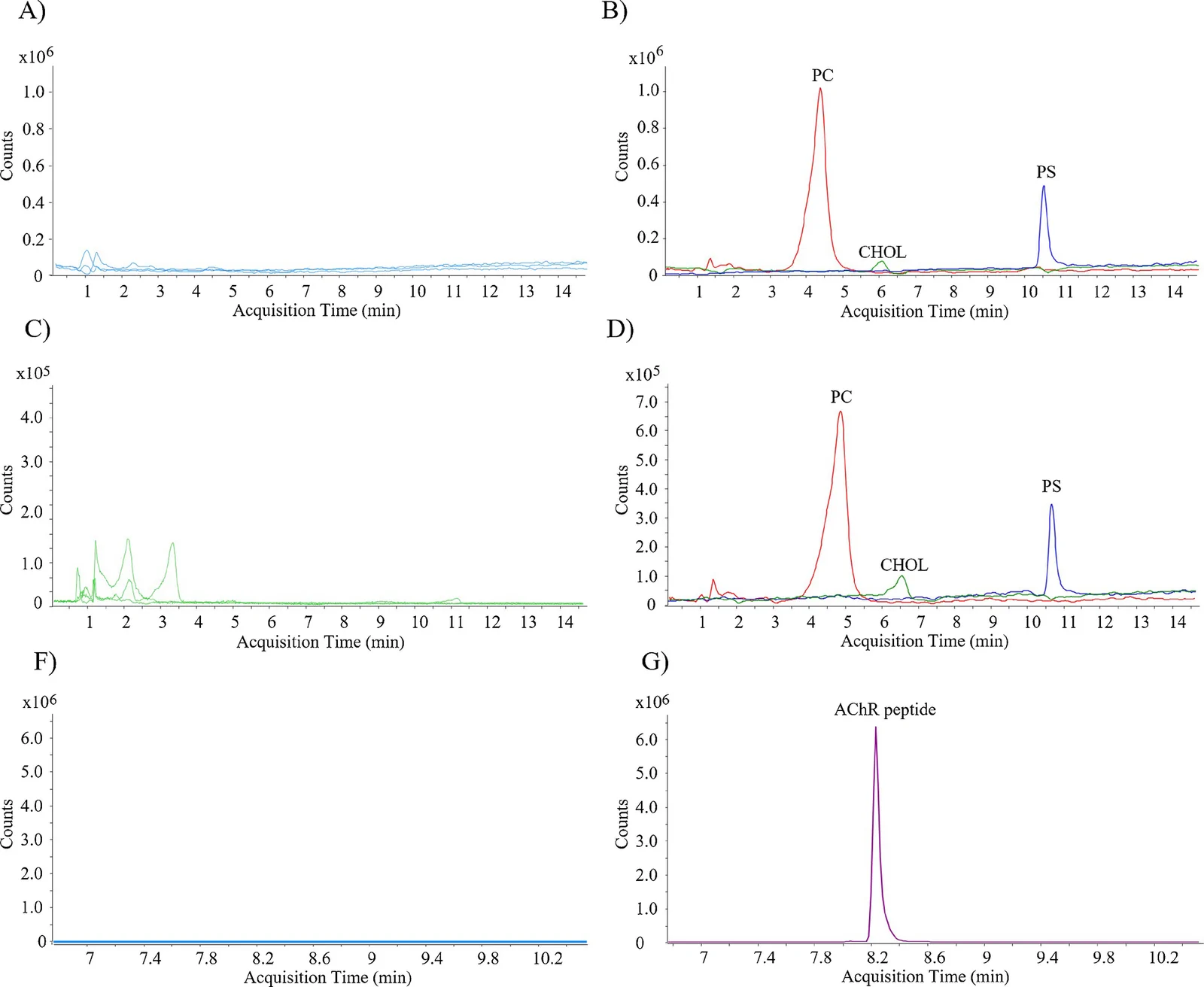
Extracted ion chromatograms (EICs) obtained by HPLC-MS for lipid analysis of **A**, **B** blank buffer solution and PS-liposome solution and **C**, **D** blank minipig serum and spiked minipig serum with PS-Liposomes, and for peptide analysis of **E**, **F** blank minipig serum and spiked minipig serum with PS-Liposomes

Once the HPLC-MS method was established, two PS-liposome formulations encapsulating AChR peptide (Batch 1 and Batch 2) at a total lipid concentration (TLC) of 1 mmol/L were purified using both qEV columns (qEV 35 and 70 nm) to compare the results with those previously obtained. The same procedure was applied to minipig serum samples spiked with PS-Liposomes at a final TLC of 1 mmol/L. In both cases, the selected fractions (4–16) were preconcentrated using the qEV kit and subjected to liquid-liquid extraction. The organic phase was then analyzed by HPLC-MS for lipid quantification using a calibration curve prepared with a standard mixture of lipids, covering a linear range of 5–25 mg/L. As shown in Table 2, when purifying PS-liposome solution, lipid recovery values determined by HPLC-MS for Batch 1 were, in general, comparable to those obtained with HPLC-ELSD (Table 1). On the other hand, both qEV columns exhibited similar recoveries and reproducibility (Table 2). However, for spiked serum samples with PS-Liposomes, it can be observed for both columns that the recoveries for spiked serum were higher than those obtained for PS-liposome solutions when using qEV kit for the preconcentration step. This could be explained by the fact that the qEV kit is described for the preconcentration of exosomes and other EVs from serum, and therefore this matrix would favor the interaction between the kit particles and PS-Liposomes. Considering that, overall, the recoveries obtained with the qEV 35 nm column are slightly higher and that the reproducibility in spiked serum is better, the selection of this column was confirmed for the subsequent experiments. Otherwise, the differences in the recoveries observed between Batches 1 and 2 may be attributed to differences in the PS-Liposomes size obtained in each batch. Batch 1 contains PS-Liposomes with larger particle sizes, resulting in higher values for all size distribution parameters (see Supplementary Figure S-7). In contrast, Batch 2 exhibits a significantly higher particle concentration. It should be noted that other factors that could potentially affect recovery, such as formulation composition, were discarded as very similar composition was obtained for both batches (see Supplementary Table S-4). Additionally, control samples collected prior to isolation were consistently analyzed and used to calculate recovery values, minimizing the impact of variations due to storage time or column-to-column variability. Figure 2 shows the extracted ion chromatograms (EICs) obtained by HPLC-MS for both blank buffer solution and PS-Liposomes (Fig. 2A, B), and blank and spiked serum with PS-Liposomes (Fig. 2C, D) for Batch 2 using qEV 35 nm column. As can be observed, no contribution of lipids derived from EVs or blood serum is observed in blank samples. Moreover, the applicability of the described methodology to serum samples is demonstrated, since almost identical chromatograms are obtained in both PS-Liposomes and spiked serum with PS-Liposomes.

**Table 2:** Lipid and antigenic peptide recoveries obtained by HPLC-MS for labelled PS-liposome formulation encapsulating AChR peptide and spiked minipig serum after isolation with both qEV columns and preconcentration using different methods (qEV kit or Centrisart filters)

Recovery (%)		qEV 35 nm column + qEV kit preconc		qEV 70 nm column + qEV kit preconc		qEV 35 nm column + Centrisart filters preconc	
		**PS-Liposomes**	**Spiked serum**	**PS-Liposomes**	**Spiked serum**	**PS-Liposomes**	**Spiked serum**
**Batch 1**	**PC**	29 ± 1	56 ± 3	26 ± 1	40 ± 3	68 ± 2	67 ± 5
	**PS**	14 ± 1	37 ± 1	13 ± 3	25 ± 8	50 ± 3	19 ± 4
	**CHOL**	32 ± 4	59 ± 1	27 ± 6	32 ± 2	67 ± 1	101 ± 17
	**TLC**	25 ± 2	51 ± 1	22 ± 1	32 ± 3	62 ± 1	62 ± 9
	**Antigenic peptide**	43 ± 1	58 ± 1	37 ± 1	49 ± 1	79 ± 2	52 ± 4
**Batch 2**	**PC**	33 ± 3	61 ± 1	31 ± 1	59 ± 6	49 ± 4	83 ± 21
	**PS**	36 ± 2	45 ± 1	34 ± 1	46 ± 3	54 ± 1	78 ± 82
	**CHOL**	60 ± 1	74 ± 1	62 ± 1	71 ± 1	94 ± 3	105 ± 35
	**TLC**	43 ± 2	61 ± 1	42 ± 1	59 ± 4	65 ± 4	89 ± 43

Subsequently, the efficacy of the two preconcentration methods previously assessed by HPLC-ELSD (qEV kit and Centrisart filters) was also studied by HPLC-MS, by using the same PS-liposome formulations (Batch 1 and Batch 2), to evaluate their performance in serum samples. With this aim, using the selected qEV 35 nm column to purify each batch, both preconcentration methods were compared. After applying the liquid-liquid extraction protocol to each sample, the obtained organic phases were analyzed by HPLC-MS. As can be observed in Table 2, the recoveries obtained for the PS-Liposomes were higher when using the Centrisart filters, as previously observed in the HPLC-ELSD analysis. On the other hand, when looking at the values in spiked serum, although the recoveries are generally higher for the Centrisart filters as well, the observed reproducibility is unsatisfactory. Moreover, the very high recoveries obtained for cholesterol (CHOL) using the Centrisart filters in spiked serum could indicate the contribution of this lipid originating from the serum. The detection of a certain amount of CHOL only when using Centrisart filters was confirmed by HPLC-MS analysis of the lipids extracted from a minipig serum blank. Thus, it was further concluded by HPLC-MS that preconcentration with the qEV kit would constitute a more suitable method for the preconcentration of PS-Liposomes isolated by SEC in blood serum samples. Otherwise, the variability observed between batches was again attributed to differences in the size distribution of the obtained PS-Liposomes, as in the previous case.

Recoveries obtained for encapsulated antigenic peptide were also studied by HPLC-MS. For this purpose, a methodology by HPLC-MS was also developed for its successful identification and quantification. By way of example, the EIC for standard acetylcholine receptor (AChR) peptide at a concentration of 10 mg/L obtained under the optimized conditions is shown in Figure S-8. Similar to lipid analysis, both qEV columns and preconcentration methods were evaluated for peptide recovery using Batch 1 as model formulation. In this case, after liquid-liquid extraction, the aqueous phase containing the encapsulated peptide was analyzed by HPLC-MS. Quantification was performed using a calibration curve prepared with high-purity peptide standard, covering a linear range of 1–20 mg/L. Table 2 shows the obtained recoveries for each condition. As can be observed, slightly higher recoveries were obtained when using qEV 35 nm column. Conversely, comparing the two preconcentration methods, Centrisart filters provided better recoveries for PS-liposome solutions while the qEV kit showed slightly higher recoveries with improved reproducibility for spiked serum. Therefore, the selection of the qEV 35 nm column, with preconcentration using the qEV kit, was also shown to be the most suitable methodology for the quantification of encapsulated peptide in blood serum samples.

Finally, in order to assess the co-isolation of serum-derived components in the pooled fractions, spiked minipig serum samples before and after isolation by qEV 35 nm only, qEV kit only, and qEV 35 nm + qEV kit method were analyzed and compared. Liquid-liquid extraction procedure was applied to each sample, and the data obtained for the analysis of each aqueous phase by HPLC-MS was processed using the MassHunter Qualitative Find Compounds by Molecular Feature algorithm to monitor serum-derived species across the different purification approaches. Using this workflow, 37 different compounds were identified when any isolation procedure is applied while only the encapsulated peptide was identified when qEV 35 nm column + qEV kit are used. Otherwise, 22 and 6 species were identified when using only qEV kit or qEV 35 nm column, respectively. These findings further confirm the effective isolation of PS-Liposomes when applying the developed methodology. Additionally, one of the detected serum-derived peptides (1858 Da; m/z observed = 930.40, *z* = 2) was selected as a target marker for serum carryover assessment. It was demonstrated that this compound originates from an endogenous minipig serum component as it was not identified when PS-liposome’s solution was analyzed. This ion was detected in spiked serum samples purified using only qEV 35 nm column or qEV kit, although with a markedly reduced signal intensity with respect to the non-purified minipig serum (see Supplementary Figure S-9). Notably, this feature was no longer detected when both qEV 35 nm column and qEV kit were combined, indicating enhanced removal of endogenous serum components. These results support the suitability of the combined qEV 35 nm column + qEV kit workflow for efficient purification of the PS-Liposomes and removal of serum-derived species.

### Application to unlabelled PS-Liposomes

Finally, the optimal conditions for the isolation of PS-Liposomes from minipig blood serum samples, and subsequent quantification of their components, by using qEV 35 nm column and qEV kit were applied to non-labelled PS-liposome formulation. It should be stated that the methodology was specifically developed for use in preclinical and toxicology studies conducted in minipigs. This model was selected because minipigs are a well-established large animal model in drug development and safety assessment, showing physiological, metabolic, and anatomical similarities to humans. The applicability of the developed method to non-labelled PS-Liposomes is important to guarantee that similar results can be obtained in pharmacokinetic and toxicology studies. To this end, a minipig serum sample spiked with a PS-liposome formulation encapsulating AChR peptide (Batch 3) at a final TLC of 1 mmol/L was subjected to the previously described isolation methodology. The purified sample was then submitted to the liquid-liquid extraction, and the resulting organic and aqueous phases were analyzed by HPLC-MS for lipids and encapsulated antigen quantification, respectively. The obtained recoveries in both cases are summarized in Table 3. As shown, very similar recoveries to those previously achieved for labelled PS-Liposomes were obtained for both lipids and encapsulated peptide (Table 2). These results pointed out the reproducibility and successful application of the established methodology for the isolation and quantification of real PS-liposome formulations intended for MG treatment.

**Table 3:** Lipid and antigenic peptide recoveries obtained by HPLC-MS for spiked minipig serum with unlabelled PS-liposome formulation encapsulating AChR peptide after isolation and preconcentration using qEV 35 nm column and qEV kit, respectively

Recovery (%)	Spiked serum
PC	42 ± 1
PS	67 ± 8
CHOL	57 ± 1
TLC	54 ± 3
Antigenic peptide	59 ± 5

**Fig. 3:**
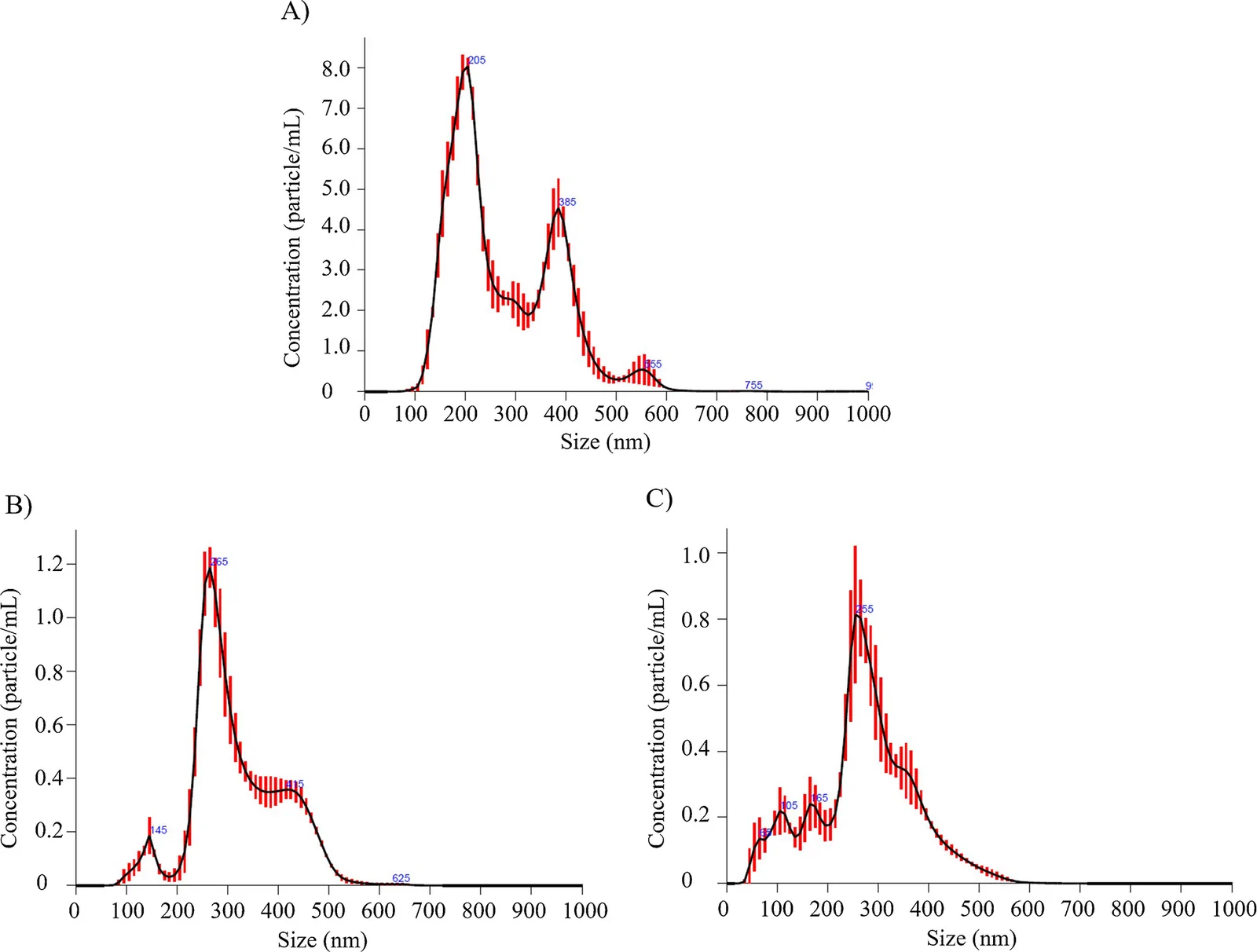
Particle concentration versus size profiles obtained by NTA for **A** initial PS-liposome formulation (control) and **B** PS-Liposomes or **C** spiked serum with PS-Liposomes after applying the established isolation methodology

**Fig. 4:**
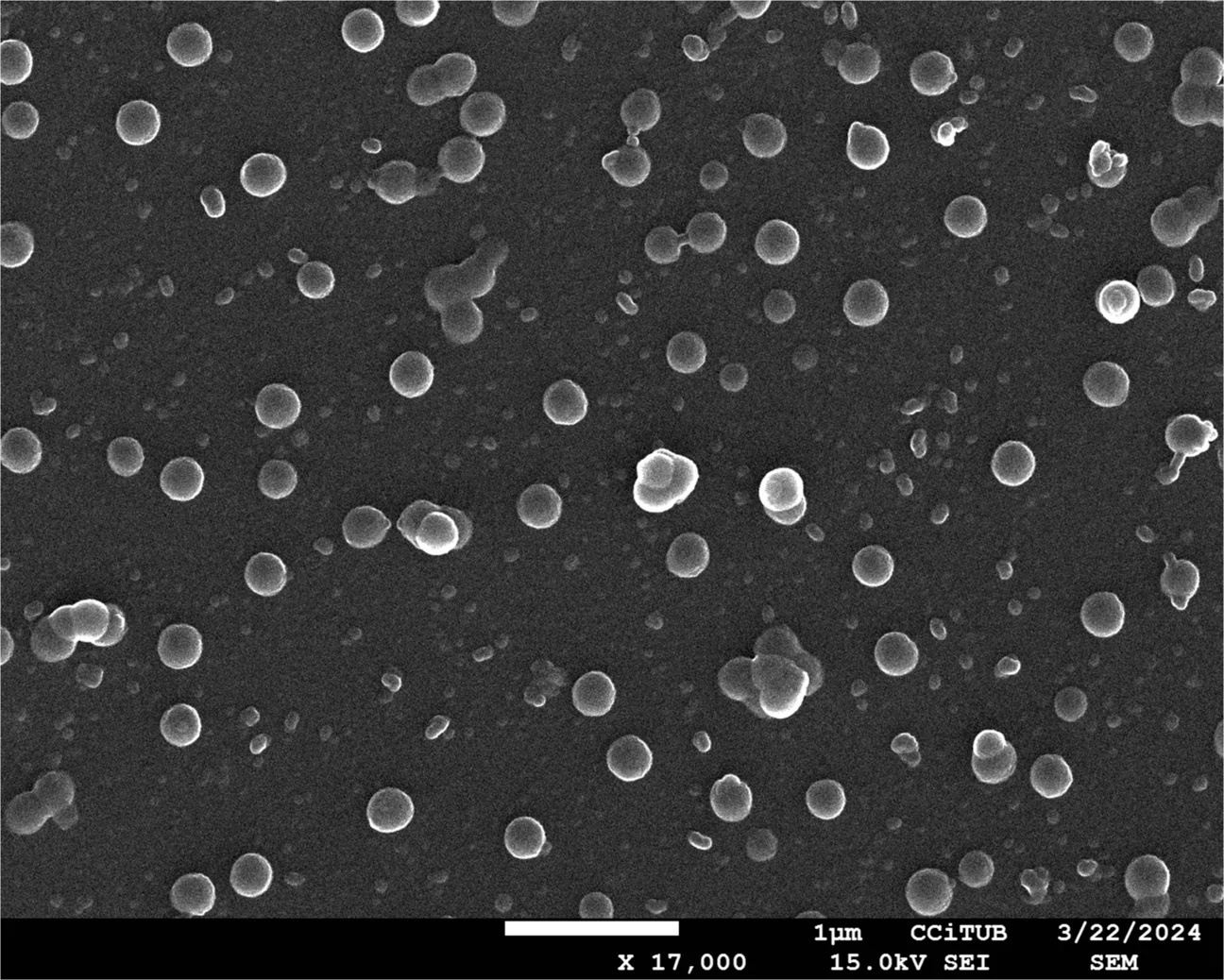
Image obtained by SEM for spiked serum with PS-liposome formulation after applying the established isolation methodology

In addition, it was decided to evaluate that the integrity and size distribution of PS-Liposomes were maintained after applying the established methodology. To this end, after isolation of PS-Liposomes and spiked serum (Batch 3), the collected fractions were preconcentrated and analyzed by two different techniques, nanoparticle tracking analysis (NTA) and scanning electron microscopy (SEM). PS-liposome formulation (Batch 3) was also analyzed as a control prior to SEC isolation. Regarding the NTA results, this technique allowed the study of the size distribution of PS-Liposomes before (control) and after purification, as well as the particle concentration obtained in each case. As shown in Table S-5, both PS-Liposomes and spiked serum exhibit very similar values to the control. However, examining the particle size distribution graphs (Fig. 3), the concentration of particles in the 100–200 nm range decreased after isolation of both PS-Liposomes and spiked serum. In contrast, the concentration of particles between 200 and 500 nm increased. Additionally, large particles (i.e., > 700 nm) are not detected after isolation. Regarding total concentration, similar values were obtained in all cases. It should be noted that NTA can show apparent differences in the concentration of size ranges when comparing samples that contain large particles with those that do not, even if the true concentration of small particles is similar. This occurs because larger particles scatter much more light and can dominate the field of view, reducing the detection and tracking of smaller particles. In addition, NTA has a limited dynamic range, and camera and detection settings optimized to visualize large particles often decrease sensitivity for smaller ones. As a result, the presence of large particles can lead to an underestimation of small-particle concentrations, meaning that observed differences may reflect in some cases technical limitations. This fact could explain the reduction of particle concentration between 100 and 200 nm in after isolation samples. Otherwise, the reduction of large particles after isolation is also expected due to the optimal recovery range (35–350 nm) of the qEV 35 nm columns employed. Considering the inherent limitations of the NTA technique and the high size polydispersity of PS-Liposomes, the observed variations were deemed acceptable, and the purified samples were considered representative of the PS-liposome formulation. Otherwise, SEM allowed obtaining images of the isolated PS-Liposomes to confirm that they preserved the structure and morphology. Figure 4 shows, as an example, a SEM image of the PS-Liposomes isolated from the spiked serum. As can be observed, liposomes maintain their integrity and spherical shape after SEC purification. In addition, the image displays a fairly homogeneous appearance, suggesting an efficient isolation of the PS-Liposomes from the proteins and aggregates present in the serum. Although this technique alone does not allow unequivocal discrimination between PS-Liposomes and extracellular vesicles (EVs), SEM images show predominantly spherical vesicular structures with sizes consistent with PS-Liposomes.

Finally, the analytical workflow for PS-liposome isolation and subsequent analysis was validated in terms of precision, accuracy, and linearity at a selected liposome concentration level (TLC 1 mmol/L). Precision was evaluated by assessing intra-day and inter-day repeatability, demonstrating consistent performance across the overall purification-analysis process. Linearity was assessed using PS-liposome formulations covering a TLC range of 0.1–2.0 mmol/L. A linear response was observed between 0.2 and 2.0 mmol/L, corresponding to the relevant concentration range for the intended application. As summarized in Supplementary Table S-6, all components showed adequate linear correlations (*R*^2^ ≥ 0.99) within the validated range. Good intra-day and inter-day precision (*n* = 3) was also achieved. Moreover, satisfactory accuracy of the overall purification and analysis procedure was obtained.

## Conclusions

In this work, the development of an analytical methodology for the isolation and subsequent characterization of PS-Liposomes encapsulating AChR antigenic peptide from minipig serum sample was described. PS-liposome’s isolation was firstly assessed by size exclusion chromatography (SEC) using different columns. A home-packed Sepharose 2B-CL column and two commercial columns with agarose resin (qEV 35 nm and qEV 70 nm) were tested by using fluorescently labelled PS-Liposomes. The obtained fractions were monitored by flow cytometry to select those containing PS-Liposomes. As a result, agarose qEV columns were selected for next experiments as they require fewer fractions to recover the main eluted PS-Liposomes. Centrisart filters and qEV concentration kit were then evaluated for PS-liposome preconcentration in the obtained SEC fractions. Lipid recoveries obtained in each case were determined by both HPLC-ELSD and HPLC-MS after liposome lysis and components extraction. Additionally, encapsulated antigen recovery was also evaluated by HPLC-MS. Results showed better performance for qEV 35 nm column in combination with qEV kit preconcentration. Finally, the established methodology was successfully applied to spiked minipig serum with unlabelled PS-liposome’s formulation. Nanoparticle tracking analysis (NTA) and scanning electron microscopy (SEM) confirmed the preservation of liposome structural integrity and circular shape after isolation and preconcentration. Nevertheless, certain loss of smaller liposomes (100–200 nm) was observed. This method provides a powerful, selective, and reproducible platform for the bioanalytical characterization of PS-Liposomes or other liposome-based drugs in biological fluids.

## Supplementary Information

Below is the link to the electronic supplementary material.

Supplementary file1 (PDF 636 KB)

## Data Availability

The authors confirm that the data supporting the findings of this study are available within the article and its Supplementary material. Raw data that support the findings of this study are available from the corresponding author upon reasonable request.
