# Supplementary material for: SEC-based isolation of phosphatidylserine-liposomes from biological samples for preclinical studies
Source: Anal Bioanal Chem. 2026 Mar 16;418(18):5965–77. doi: 10.1007/s00216-026-06436-y (PMC13541980; doi:10.1007/s00216-026-06436-y)
Supplement: Supplementary file 1 [file 216_2026_6436_MOESM1_ESM.pdf]

SUPPLEMENTARY MATERIAL

SEC-Based Isolation of Phosphatidylserine-Liposomes from Biological Samples for Preclinical Studies

Oumaima El Ouahabi[1,2], Montserrat Mancera-Arteu[1,*], Ángeles López-Godoy[2], Irene Latorre[1,2], Míriam Salvadó[2], Bruna Barneda[2], Sílvia Rodríguez-Vidal[2], Victoria Sanz-Nebot[1]

[1]Department of Chemical Engineering and Analytical Chemistry, Institute for Research on Nutrition and Food Safety (INSA·UB), University of Barcelona, Martí i Franquès 1-11, 08028 Barcelona, Spain

[2]Ahead Therapeutics SL, Barcelona, Spain

*Corresponding author: montsemancera@ub.edu (M. Mancera-Arteu, PhD)

Supplementary Experimental

1. Flow Cytometry

Fractions obtained from size-exclusion chromatography (SEC) were analyzed by flow cytometry using Gallios multi-color Flow Cytometer (Beckman Coulter, Brea, CA, USA) equipped with three lasers (red, blue, and violet) in a standard 10-color configuration. PS-liposomes labeled with NBD-cholesterol were diluted 1:5 in PBS prior to acquisition, and fluorescence was recorded on the FL1 channel (Discriminant 1, 500 V) under logarithmic scale settings. For particle counting control, a suspension of Flow-Check fluorescent beads of known concentration was prepared by mixing 50 µL of the stock solution with 450 µL of PBS. This standard was used to verify instrument performance and ensure accurate quantification of PS-liposome events.

2. Nanoparticle Tracking Analsys (NTA)

PS-liposome size distribution and particle concentration were measured using a Nanosight NS300 system (Malvern Panalytical, Malvern, UK) equipped with a Blue488 laser and sCMOS camera. Samples were diluted with DPBS 1:100 to fall within the working range (10^6–10^9 particles/mL). The samples were loaded into the sample chamber at 17.9 °C. Three videos were acquired for each sample. Videos were analyzed with Nanosight NS300 software (Version 3.4) with detection threshold 5, blur size auto, and maximum jump distance 10.1–10.4 pixels to determine particle size and concentration.

3. Scanning electron microscopy (SEM)

For scanning microscope analysis (SEM), samples were fixed with 2.5% glutaraldehyde in 0.1M phosphate buffer and placed on poly-lysine coated coverslips. They were then post-fixed with 1% osmium tetroxide in the same phosphate buffer, dehydrated through a graded alcohol, and processed for critical point drying using an Emitech K850. A thin carbon coating was applied to enhance electrical conductivity. Samples were examined with Jeol JSM-7001F (Jeol, Japan) operated at 15 kV at the TEM-SEM Electron Microscopy Unit of Scientific and Technological Centers (CCiTUB), University of Barcelona.

A)

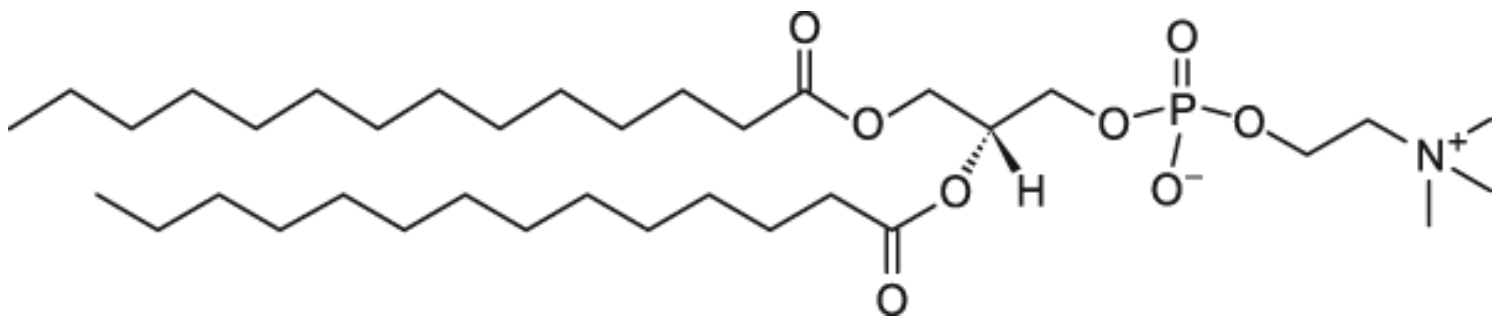

B)

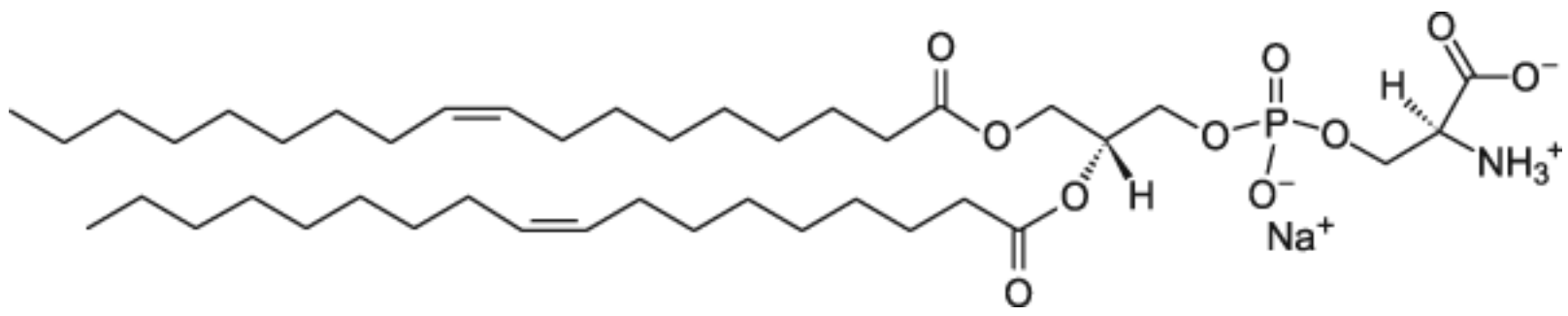

C)

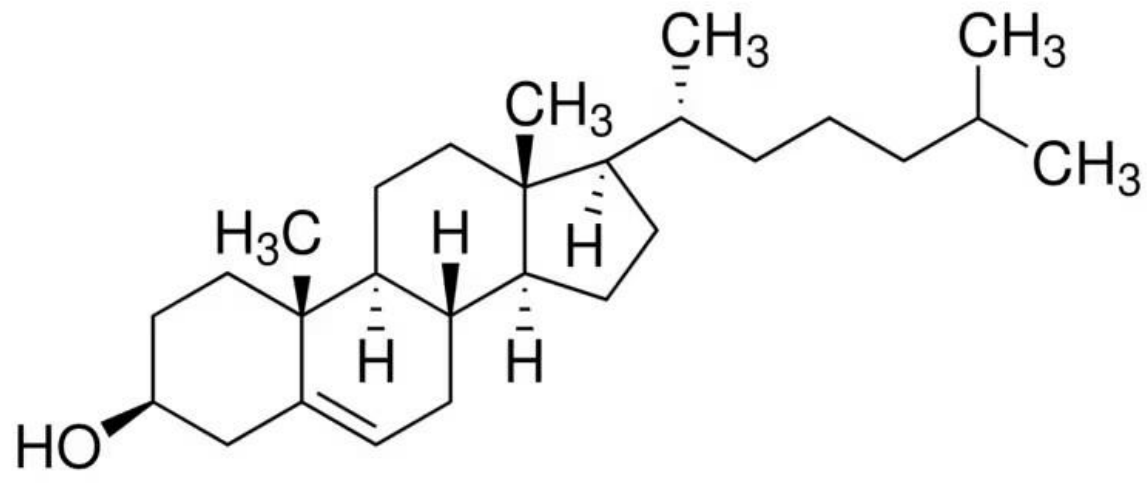

Figure S-1. Chemical structures of the lipid standards used in this study. A) Phosphatidylcholine (PC, 14:0, MW 677.94 g/mol) B) phosphatidylserine (PS, 18:1, MW 810.04 g/mol). C) cholesterol (MW 386.65 g/mol).

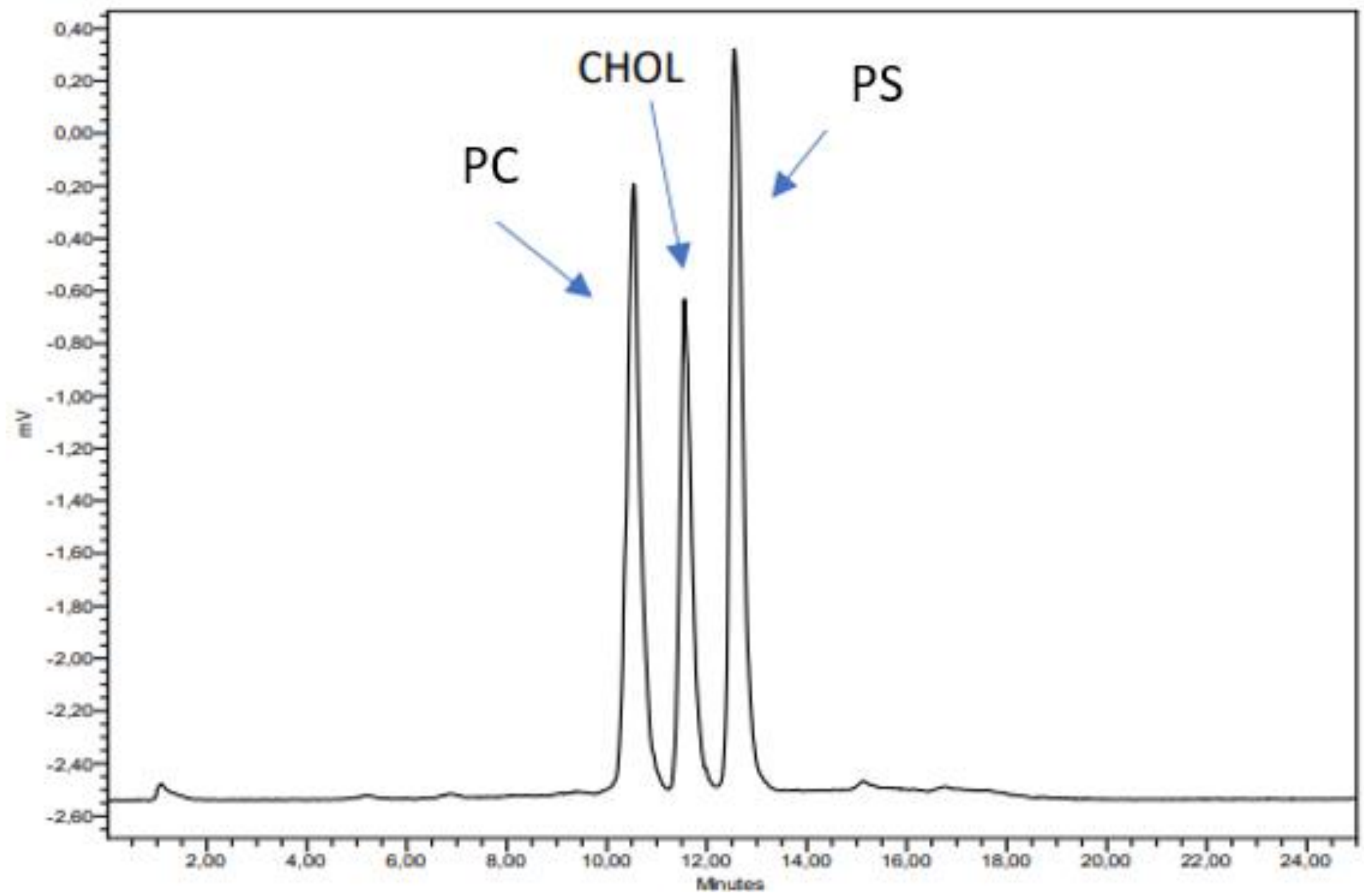

Figure S-2.- Chromatogram obtained by HPLC-ELSD for lipids standard mixture (PC, PS and CHOL) at a concentration of 200 mg/L each one.

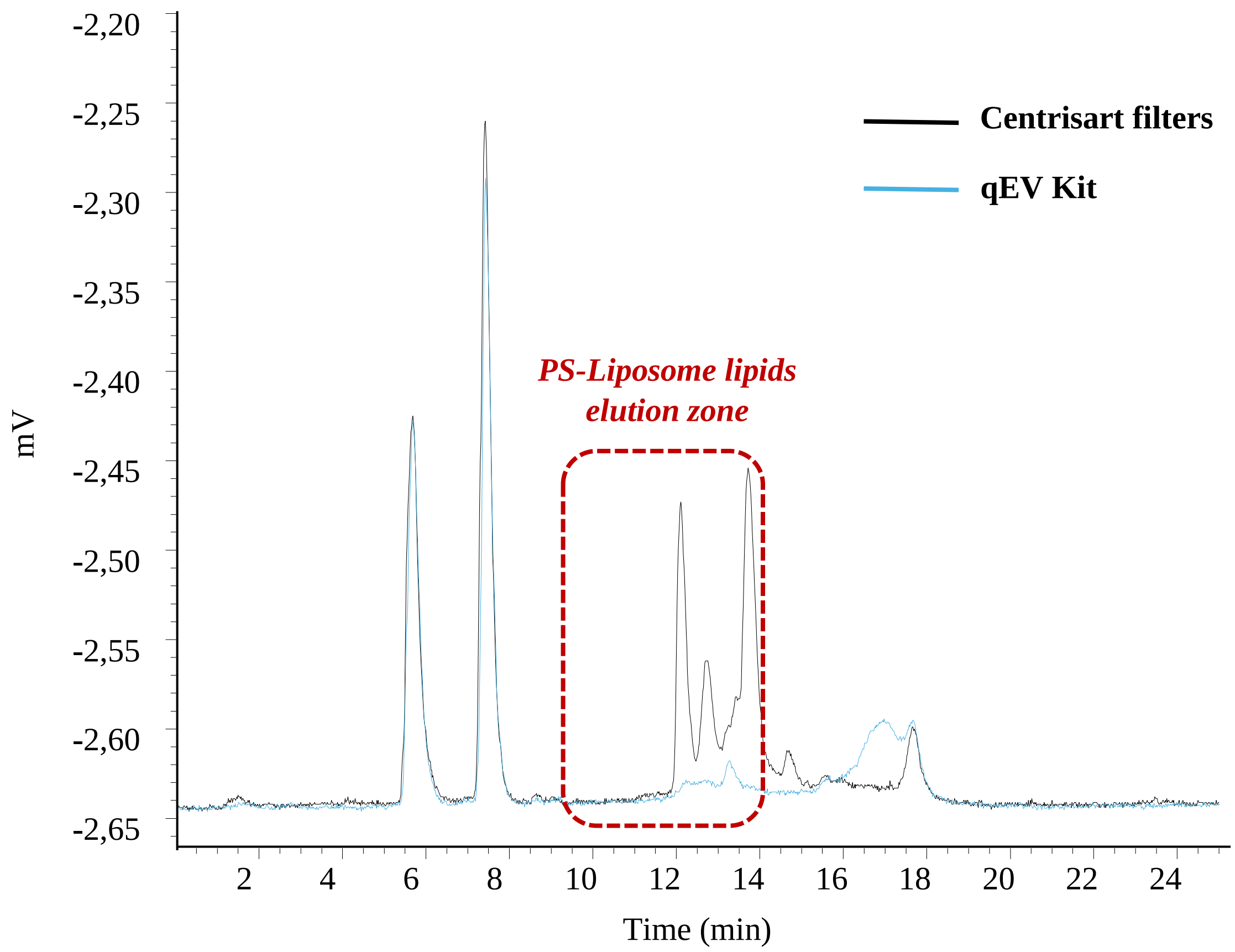

Figure S-3.- Chromatograms obtained by HPLC-ELSD for blank minipig serum samples purified using qEV 35 nm column, followed by a preconcentration with Centrisart filters or qEV kit. Lipids from PS-Liposomes elute in the region between 10 and 14 minutes.

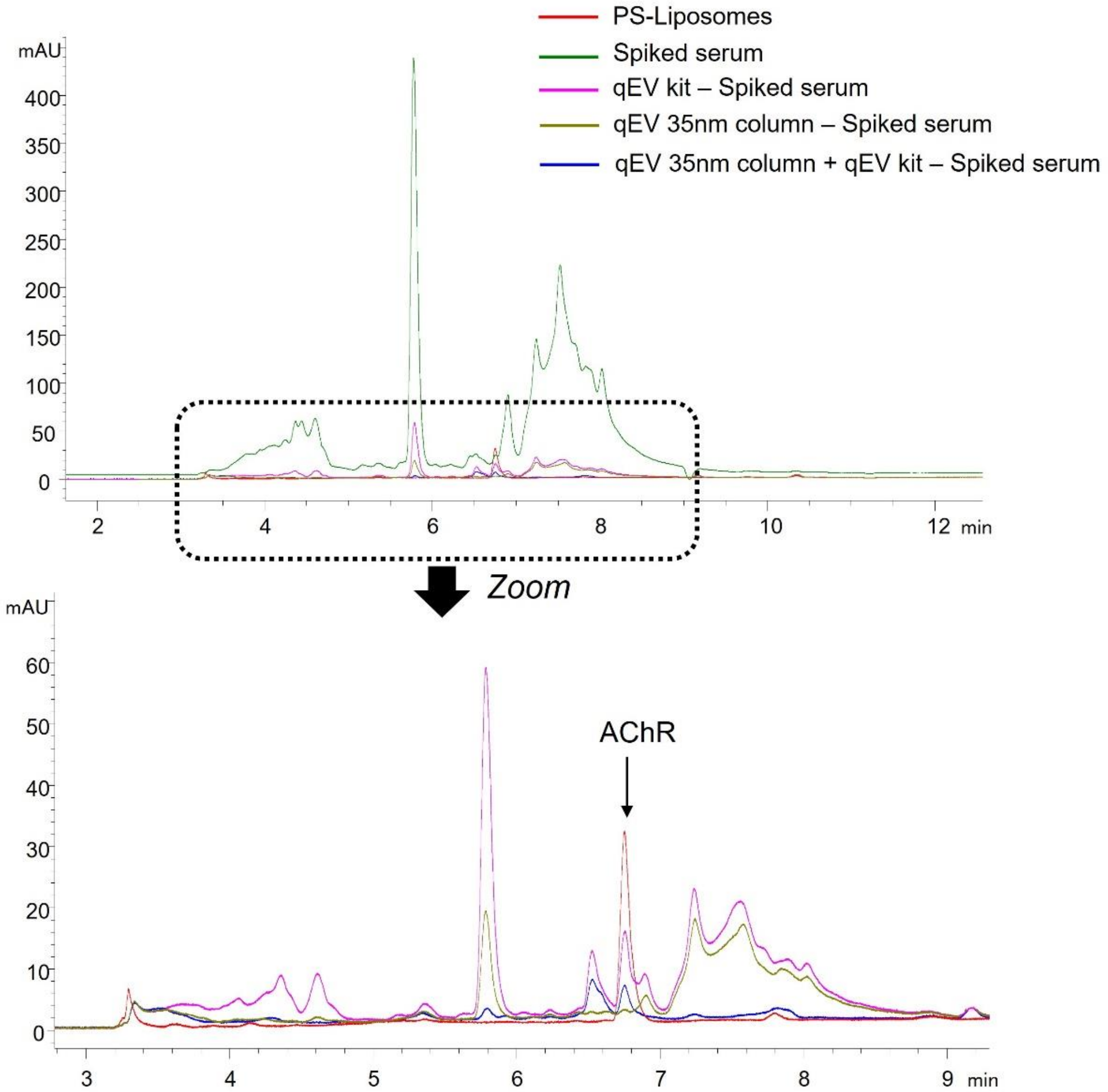

Figure S-4.- Chromatograms obtained by HPLC-UV for the analysis of the aqueous phases after applying the liquid-liquid extraction procedure to spiked minipig serum samples with PS-Liposomes before and after isolation by qEV 35 nm only, qEV kit only, and qEV 35nm + qEV kit method.

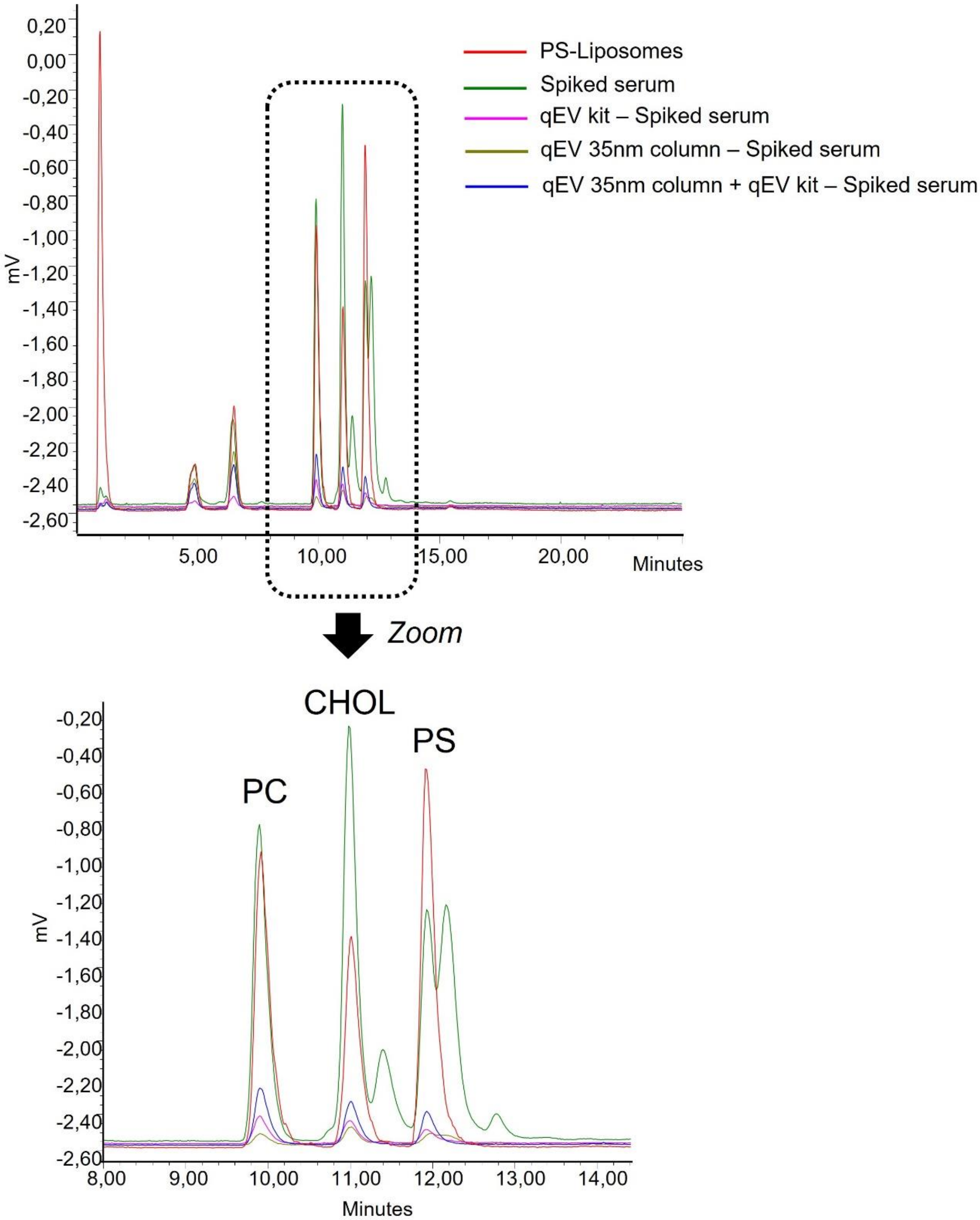

Figure S-5.- Chromatograms obtained by HPLC-ELSD for the analysis of the organic phases after applying the liquid-liquid extraction procedure to spiked minipig serum samples with PS-Liposomes before and after isolation by qEV 35 nm only, qEV kit only, and qEV 35nm + qEV kit method.

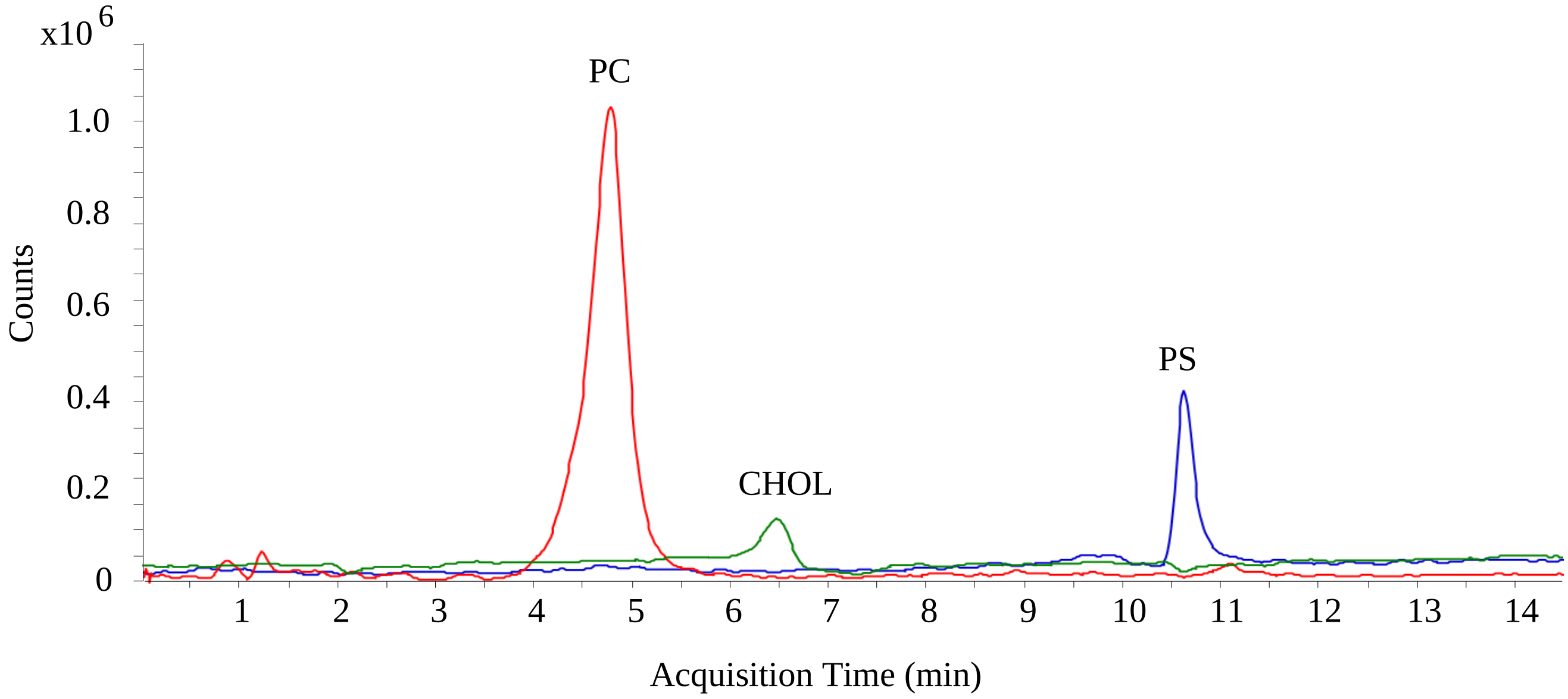

Figure S-6.- Extracted ion chromatograms (EICs) obtained by HPLC-MS for lipids standard mixture (PC, PS and CHOL) at a concentration of 25 mg/L each one.

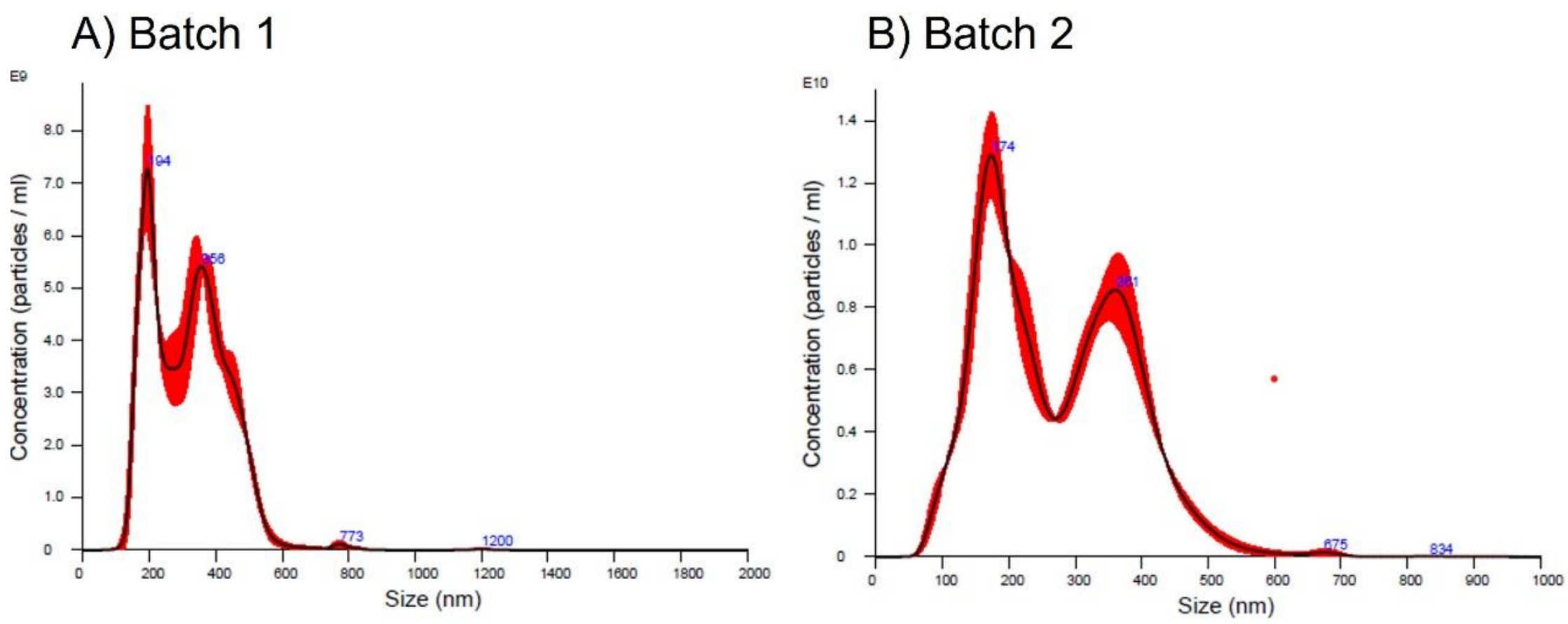

Sample	Average (nm)	D50 (nm)	D90 (nm)	Concentration ($x\ 10^{12}$, part/mL)
Batch 1	318 ± 1	318 ± 4	467 ± 2	1.62 ± 0.04
Batch 2	273 ± 2	258 ± 5	414 ± 5	2.58 ± 0.01

Figure S-7.- Particle concentration versus size profiles obtained by NTA for (A) Batch 1 and (B) Batch 2, and the obtained size distribution and particle concentration results.

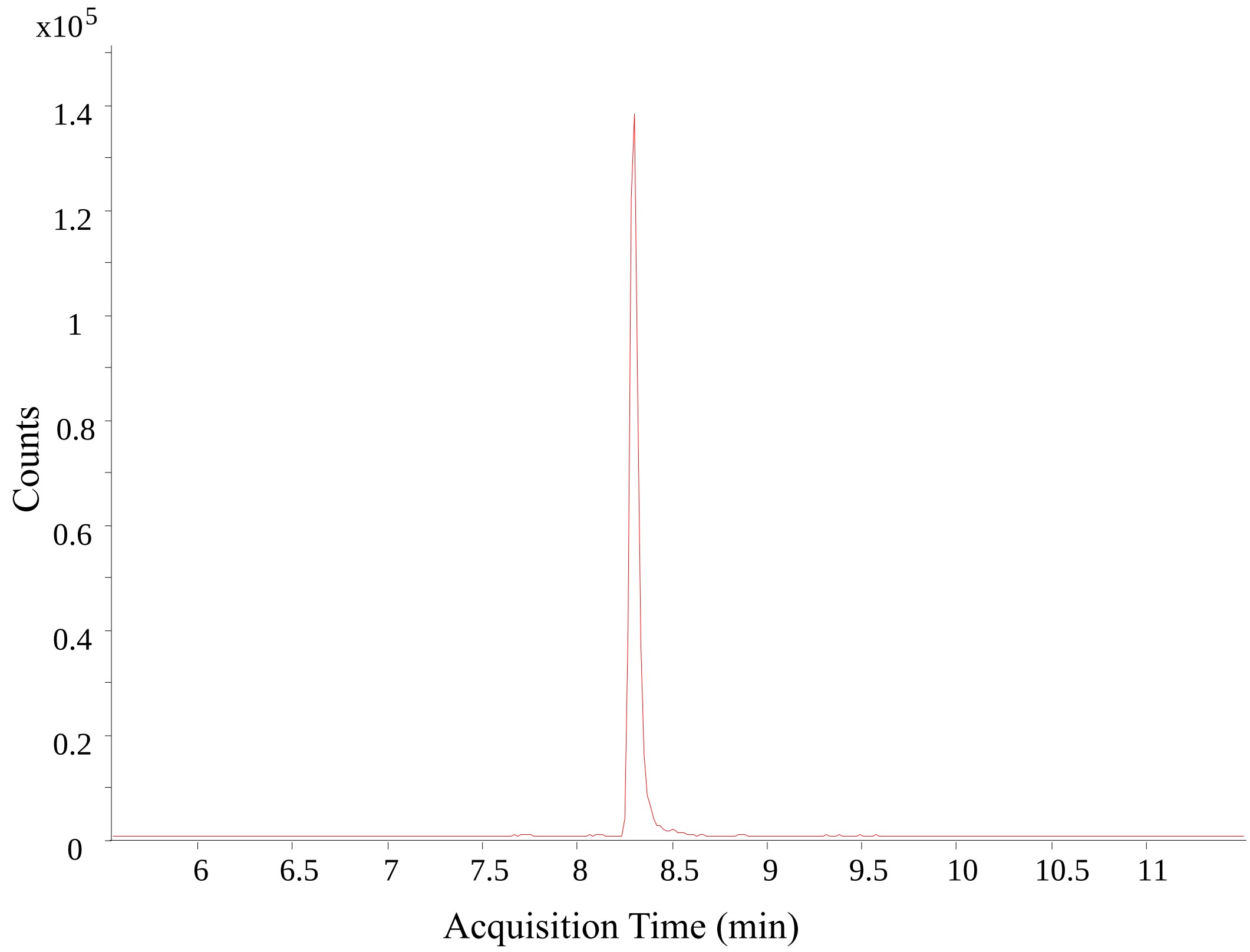

Figure S-8.- Extracted ion chromatogram (EIC) obtained by HPLC-MS for standard acetylcholine receptor (AChR) peptide at a concentration of 10 mg/L.

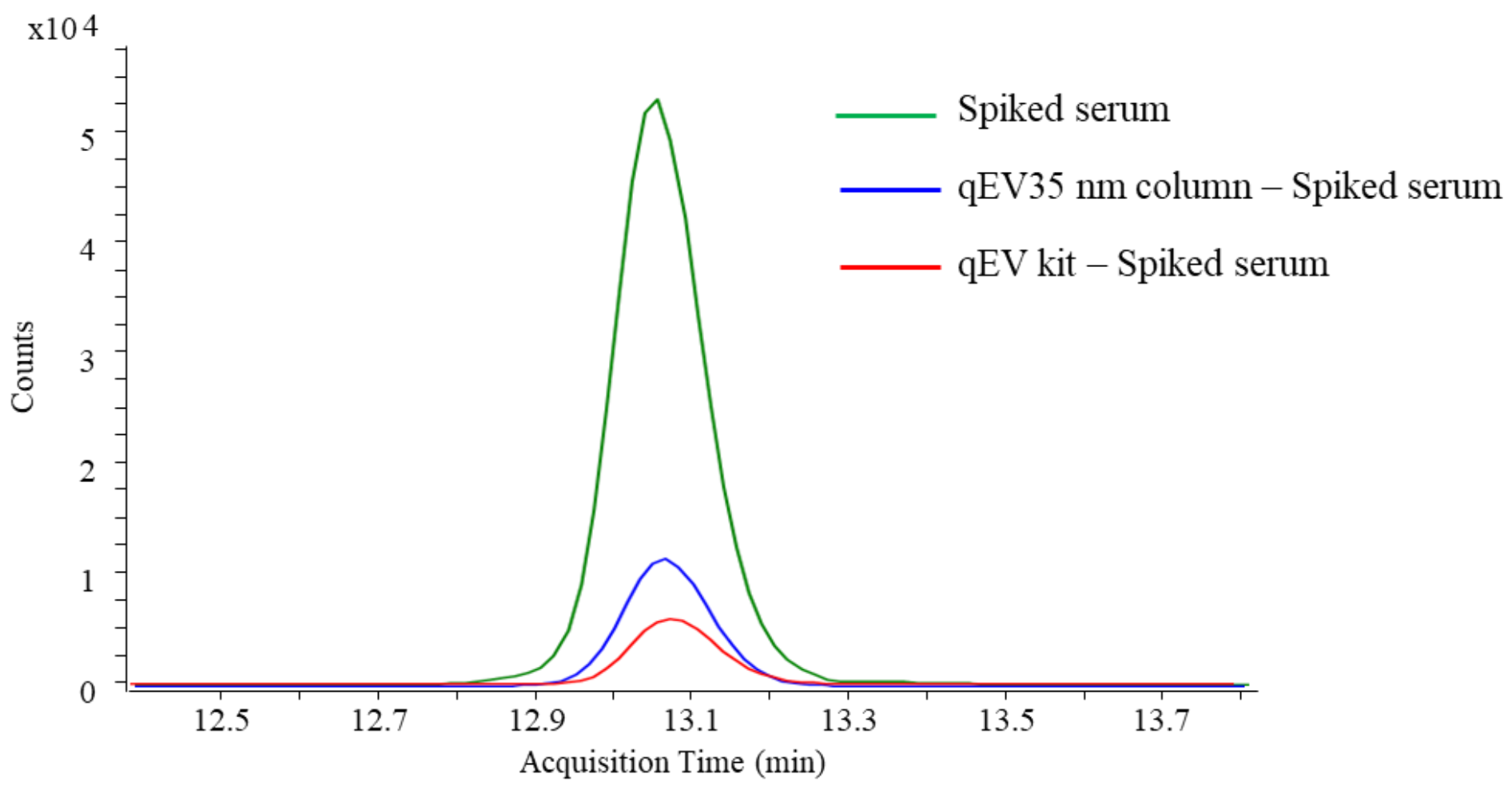

Figure S-9.- Extracted ion chromatograms (EIC) obtained by HPLC-MS for the serum-derived peptide (1858 Da; m/z observed = 930.40, z = 2), selected as a target marker for

serum carryover assessment, in spiked minipig samples with PS-Liposomes before and after isolation with qEV 35 nm column or qEV kit.

Table S-1.- Target ions and exact m/z values used for HPLC-MS quantification of PC, PS, cholesterol and AChR peptide.

Analyte	Ion monitored	Exact m/z	Ionization mode	Mass tolerance
PC	$[M+H]^+$	678.5074	ESI+	±10 ppm
PS	$[M+H]^+$<br>$[M-Na+2H]^+$	810.5260<br>788.5442	ESI+	±10 ppm
Cholesterol	$[M+Na]^+$	409.3446	ESI+	±10 ppm
AChR peptide	$[M+2H]2^+$	1091.5279	ESI+	±10 ppm

Table S-2.- Lipid recoveries obtained by HPLC-ELSD for labelled PS-Liposomes formulation encapsulating AChR peptide after isolation and preconcentration using qEV 35 nm column and qEV kit, respectively, collecting different SEC fractions.

**Recovery (%)**		
	**F4-16**	**F4-24**
**PC**	45	43
**PS**	39	34
**CHOL**	49	47
**TLC**	44	42

Table S-3.- Lipid recoveries obtained by HPLC-ELSD for labelled PS-liposomes formulation at different concentrations, after isolation and preconcentration using qEV 35 nm column and qEV kit, respectively.

Concentration (mmol/L)	Recovery (%)			
	PC	PS	CHOL	TLC
0.2	18	28	18	20
0.5	27	20	25	24
1	36	29	34	33
2	34	30	36	34

Table S-4.- Concentrations (mg/L) determined for all lipids that conforms the membrane and, free and encapsulated antigenic peptide in the studied PS-Liposome batches.

Concentration (mg/L)		Batch 1	Batch 2	Batch 3
Lipids	PC	6104.8	6318.1	6857.1
	PS	7926.4	7720.4	7458.5
	CHOL	4267.0	3919.2	4424.9
	TLC	18298.2	17957.7	18740.5
Antigenic peptide	Free	19.8	20.5	19.3
	Encapsulated	300.4	313.8	292.5

Table S-5.- Size distribution and particle concentration results obtained by NTA for unlabelled PS-liposomes formulation encapsulating AChR peptide and spiked minipig serum, before (control) and after isolation. D50 and D90 parameters represent the particle diameters at which 50% and 90% of the population, respectively, exhibit smaller sizes.

Sample	Average (nm)	D50 (nm)	D90 (nm)	Concentration ($x 10^{11}$, part/mL)
Control	276 ± 4	241 ± 6	414 ± 5	1.25 ± 0.02
PS-Liposomes	317 ± 1	297 ± 1	442 ± 2	1.51 ± 0.05
Spiked Serum	272 ± 4	273 ± 4	397 ± 4	1.28 ± 0.02

Table S-6.- Linearity, precision and accuracy evaluation of the PS-Liposomes purification workflow using HPLC-MS analysis.

**Analyte**	**Linearity Y = AX + B (R^2) (0.2-2 mmol/L)**	**Intra-day precision (%RSD) (n=3)**	**Inter-day precision (%RSD) (n=3)**	**Recovery (%)**
**PC**	y = 0.4x – 8.5 (0.993)	4.6	7.6	43.1 ± 3.5
**PS**	y = 0.2x – 7.6 R^2 = (0.995)	2.0	6.3	43.5 ± 3.1
**CHOL**	y = 0.4x – 6.1 (0.993)	5.4	7.2	31.1 ± 1.7
**AChR peptide**	y = 0.3x + 0.1 (0.994)	5.9	9.0	66.5 ± 7.2
